# CRISPR-Cas9 mediated CD133 knockout inhibits colon cancer invasion through reduced epithelial-mesenchymal transition

**DOI:** 10.1371/journal.pone.0220860

**Published:** 2019-08-08

**Authors:** Wanlu Li, Mee-Yon Cho, Suji Lee, Mirae Jang, Junsoo Park, Rackhyun Park

**Affiliations:** 1 Department of Pathology, Yonsei University Wonju College of Medicine, Wonju, South Korea; 2 Division of Biological Science and Technology, Yonsei University, Wonju, South Korea; University of South Alabama Mitchell Cancer Institute, UNITED STATES

## Abstract

We previously reported that CD133, as a putative cancer stem cell marker, plays an important role in cell proliferation and invasion in colon cancer. To understand the role of CD133 expression in colon cancer, we evaluated the inhibitory effect of CD133 in colon cancer cells. In this study, we generated CD133^knockout^ colon cancer cells (LoVo) using the CRISPR-Cas9 gene editing system. CD133^+^ colon cancer cells (LoVo) were infected with the lentiviral vector carrying CD133 gRNA and purified cell by culturing single cell colonies. CD133^knockout^ cells was validated by western blot and flow cytometry analysis. In functional study, we observed a significant reduction in cell proliferation and colony formation in CRISPR-Cas9 mediated CD133 ^knockout^ cells in compare with control (P < 0.001). We also found the anticancer effect of stattic was dependent on CD133 expression in colon cancer cells. Although CD133^knockout^ cells could not completely block the tumorigenic property, they showed remarkable inhibitory effects on the ability of cell migration and invasion (P < 0.001). In addition, we examined the epithelial mesenchymal transition (EMT)-related protein expression by western blot. The result clearly showed a loss of vimentin expression in CD133^knockout^ cells. Therefore, CRISPR-Cas9 mediated CD133^knockout^ can be an effective treatment modality for CD133^+^ colon cancer through reducing the characteristics of cancer stem cells.

## Introduction

Cancer stem cells (CSCs) are considered to be small subsets of tumor cells which exhibit stem-cell-like characteristics and are closely related to self-renewal, tumorigenesis, multi-directional differentiation potential, metastasis initiation, and therapeutic resistance [1]. Identification of different subgroups of CSCs by using the expression of cell surface antigens (surface markers) is the most convincing method today [2]. Furthermore, these cancer stem cell markers play an important role in biological functions [3]. CD133, a putative CSC marker, has been receiving much attention as a candidate for target therapy [4, 5]. CD133 expression was reported to be related with poor prognosis, metastasis, and recurrence in colon cancer [6]. However, controversial results have been reported that both CD133^+^ and CD133^-^ cells could initiate colon cancer [7]. In our previous study, we found that inhibition of cell proliferation after CD133 siRNA transfection was closely related to decreased survivin expression [8, 9]. Recently, we found activated signal transducer and activator of transcription 3 (STAT3) is upregulated by interleukin (IL)-6 and blocking of the STAT3/CD133/survivin signaling pathway showed anticancer effect in colon cancer *in vitro* [8, 10]. However, the mechanism involved in CD133 deficiency and changes in invasive ability in cancer cells was not described. Epithelial-mesenchymal transition (EMT), as a misactivation of the embryonic dedifferentitation program, is a critical process in malignant progression [11]. Epithelial cells lose polarity, resulting in decreased adhesion and enhanced migration or invasion [11]. Turano *et al*. reported atypical nuclear localization of CD133 in a majority of colon cancer cells with more mesenchymal phenotype [12].

Clustered regularly interspaced short palindromic repeats-associated nuclease 9 (CRISPR-Cas9) provides a new genome editing tool that precisely manipulates specific genomic loci and helps elucidate target gene function or disease [13]. CRISPR-Cas9 system can edit a specific gene through cutting double-strand DNA, and host cell responds to this double stand breaks by non-homologues end joining (NHEJ) or homology-directed repair (HDR) [14]. CRISPR-Cas9 mediated gene editing has been used in many cell lines and organisms and is an effective translational tool for gene editing studies [15]. To identify the essential cancer cell genes, a genome-scale CRISPR-Cas9 knockout library of the guide sequence has been established [16]. Thus, CRISPR-Cas9 system has been a powerful and efficient gene editing tool in biomedical research.

In this study, to determine the role of CD133 in colon cancer, we established a CD133^knockout^ colon cancer cell line using CRISPR-Cas9 gene editing system and analyzed whether CD133 knockout contributed to attenuate the abilities of colon cancer cells, including colony formation, cell proliferation, migration, and invasion.

## Material and method

### I. Generation of CD133^knockout^ cell line with CRISPR-Cas9 gene editing system

#### Cell line and culture

We purchased human colon adenocarcinoma LoVo cell line from Korean Cell line Bank (KCLB number 10229, Seoul, Korea). LoVo cells were cultured in Roswell Park Memorial Institute 1640 Medium (RPMI-1640) (Hyclone, Logan, Utah, USA) supplemented with 10% fetal bovine serum (Gibco, Grand Island, NY, USA), penicillin (100 U/ml), and streptomycin (100 mg/ml; Gibco) in a humidified atmosphere at 37°C and 5% CO_2_.

#### Generation of CD133 knockout cell line with CRISPR-Cas9

Guide RNA sequences for CRISPR-Cas9 were designed at CRISPR design website (http://crispr.mit.edu/). Insert oligonucleotides for human CD133 gRNA was 5’-CACCGCAACAGGGAGCCGAGTACG-3’ and 5’-AAACCGTACTCGGCTCCCTGTTGC-3’. The CD133 guide RNA targets exon 1 of the CD133 gene. The complementary oligonucleotides for guide RNAs (gRNAs) were annealed, and cloned into LentiCrispr V2 vector. Green fluorescent protein (GFP) lentivirus vector was used as a control. LentiCrispr V2 vector was transfected to HEK-293T cells using calcium phosphate and media was exchanged after 12h. The lentivirus was harvested from the media after three days, and viral particles were concentrated by centrifugation. LoVo cells were transfected with CRISPR-Cas9 lentiviruses or the GFP lentivirus control. The GFP-positive cells were counted to ensure the efficiency of transfection. The CD133 expression was analyzed by western blotting and flow cytometry analysis.

#### Culturing single cell colony

CD133^knockout^ cells were cultured in complete media and then diluted to a final concentration of 50 cells/ml. Cells were re-suspended in fresh media, and the cell suspension was transferred to a 100 mm dish. Cells were cultured for 7 days until every single cell grew to a colony. Each colony was removed by trypsin and transferred to a 24-well plate. The cells were allowed to proliferate until there were enough cells for both functional and molecular assays.

#### Flow cytometry analysis

Trypsinized cells were centrifuged and resuspended in FACS buffer (PBS, pH 7.2, containing 0.5% fetal bovine serum and 2 mM ethylenediaminetetraacetic acid). Phycoerythrin (PE)-conjugated immunoglobulin G (isotype control), and CD133/1-PE (Miltenyi Biotec, Bergisch Gladbach, Germany) were added to each group with an FcR blocking reagent (Miltenyi Biotec) and incubated in the dark for 10 min at 4°C. After washing, the cells were resuspended in FACS buffer for analysis with a BD FACS Aria III (BD Bioscience, San Jose, CA, USA).

### II. Assay for the changes of colony formation, cell proliferation, invasion and migration in colon cancer cells after knockout of CD133 using CRISPR-Cas9

#### Colony formation assay

LoVo cells and CRISPR-Cas9 induced CD133knockout cells were re-seeded into 6‑well plates at a density of 5000 cells/well and incubated for 10 days. The cell culture medium was changed every three days. At the end of each experiment, the cells were fixed by methanol for 5 min and visualized by using 1% methylene blue. The experiment was conducted in duplicate and repeated at least twice.

#### Cell proliferation assay

We used WST-1 assay to measure the cell proliferation. Cells were suspended in RPMI containing 10% FBS, seeded in 96-well plates at a concentration of 5x103 cells/well in a volume of 100 μl culture medium, and then, incubated in a humidified atmosphere at 37°C and 5% CO2. After treatment with stattic (5 μM) (Santa Cruz Biotechnology, Santa Cruz, CA, USA) or YM155 (5 nM) (Selleckchem, Houston, TX, USA) for 24 and 48 h, 10 mL WST-1 reagent (Roche, Indianapolis, IN, USA) was added and further incubated for 1 h at 37°C and 5% CO2. The absorbance of the samples was measured using an enzyme-linked immunosorbent assay (ELISA) reader at 450 nm wavelength.

#### Wound healing assay

The cells were treated with 0.1 mg/mL mitomycin C (Sigma-Aldrich) for 2 h before scratching to distinguish cell migration from proliferation [17]. The monolayer was scratched by using a 200 mL sterile pipette tip. After 24 h, cell migration was examined by microscopy and analyzed objectively using ImageJ software. The wound healing rate was calculated using the formula: wound healing rate (%) = [(width at 0 h—width at 24 h)/(width at 0 h)] x 100.

#### Transwell migration and invasion assay

Transwell chambers with 8-μm pores were obtained from Corning. Cells were harvested and resuspended in RPMI with 10% FBS at a concentration of 1 × 10^6^ cells in 1 mL, and then seeded into the upper chamber of a 12-well plate. The lower chambers were filled with 2.3 mL RPMI containing 20% FBS. Cells were incubated for 24 h. At the end of the experiment, cells that migrated into the reverse side of the transwell membrane were fixed with methanol, stained with hematoxylin and eosin stain (HE stain), and then, counted under an inverted light microscope. Transwell invasion assay was performed with a similar protocol as migration assay except that the transwell chambers were coated with Matrigel.

### III. Molecular changes of colon cancer cells after CD133 knockout using CRISPR-Cas9

#### Western blotting

We evaluated the molecular changes using the cells from 10 different colonies of single colony assay. Protein extraction and western blot procedures were described in our previous reports [8, 9]. In the current research, the antibodies used are as follows: anti-CD133/1 (AC133, Miltenyi Biotech, Bergisch Gladbach, Germany), anti-survivin (Abcam, Cambridge, MA, USA), anti-cleaved caspase-3 (Cell Signaling Technology), anti-vimentin (Dako, Denmark), anti-E-cadherin (Transduction Laboratories, Lexington, KY, USA), anti-Twist (Abcam, Cambridge, UK), CXCR4 (Abcam, Cambridge, UK) and anti-beta-actin (Santa Cruz Biotechnology, Santa Cruz, CA, USA).

#### Immunohistochemical analysis

The immunohistochemical (IHC) staining for vimentin (Dako, Denmark, 1:4000) was performed as we reported before [10].

#### Statistical analysis

All statistical analyses were performed using IBM SPSS Statistics for Windows (version 23.0; IBM Corporation, Armonk, NY, USA). Categorical variables were described by frequencies and percentages and compared using chi-square or Fisher's exact test as appropriate. Continuous variables were described as the mean ± standard deviation and were analyzed by Student's t-tests and analysis of variance (ANOVA). A p-value of <0.05 was considered significant.

## Results

### CD133 knockout using CRISPR-Cas9 gene editing system

**Fig 1:**
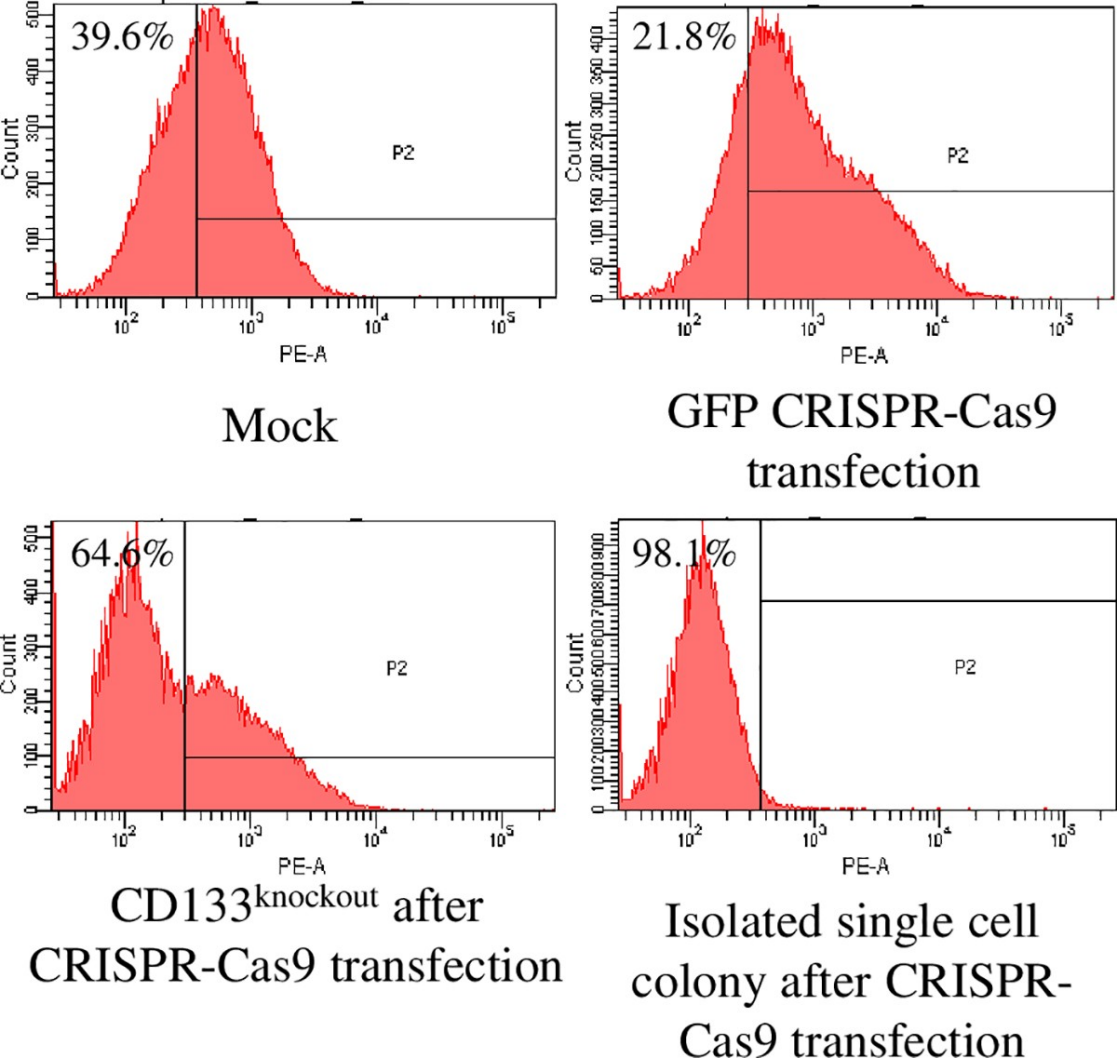
Validation of CD133 knockout by flow cytometry analysis. CRISPR-Cas9 mediated CD133^knockout^ group was composed of 64.6% CD133^-^ cells, in contrast to 39.6% and 21.8% in the mock and GFP group, respectively. After culturing isolated single cell colony from the CD133 CRISPR-Cas9 transfected cells, the proportion of purified CD133^knockout^ cells was 98.1%.

The flow cytometry assay showed that CRISPR-Cas9 mediated CD133^knockout^ group was composed of 64.6% of CD133^-^ cells, in contrast to 21.8% in the GFP group (Fig 1). The CD133^knockout^ cells were estimated to be >98% after culturing an isolated single colony of CD133^knockout^ cell (Fig 1).

### Inhibition of self-renewal and cell proliferation in CD133^knockout^ colon cancer cells were related to decreased survivin

**Fig 2:**
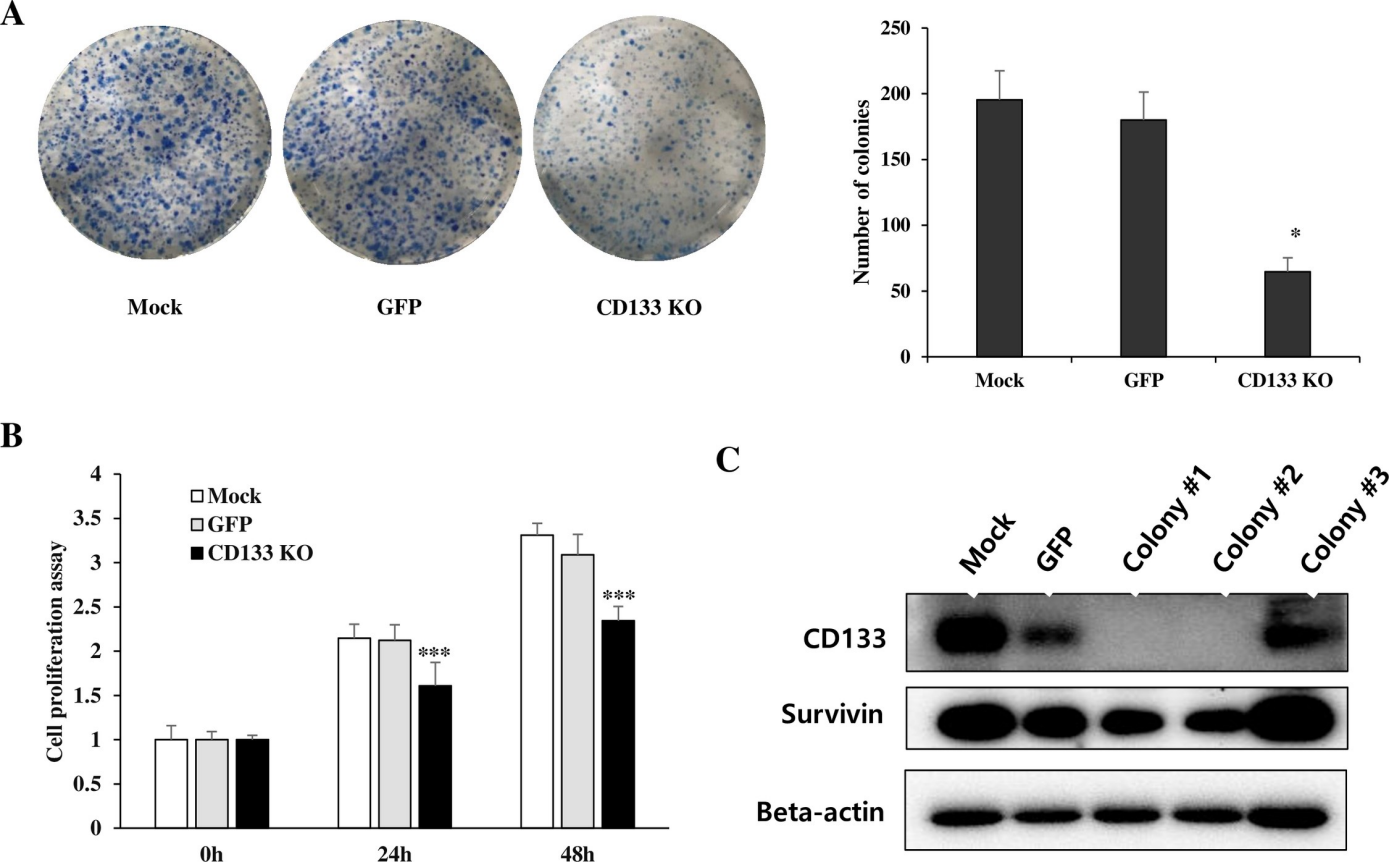
CD133 knockout inhibited cell colony formation and proliferation. (**A**) Representative photographs of the colony formation assay demonstrated that the smallest number of colonies was observed in the CD133^knockout^ group compared with mock and GFP groups (mock *vs*. CD133 KO, P = 0.017; GFP *vs*. CD133 KO, P = 0.0172; *P<0.05). (**B**) WST-1 assay also showed a significant reduction in cell proliferation of CD133^knockout^ cells compared with mock and GFP groups (***P<0.001). (**C**) In western blot, we confirmed that the CRISPR-Cas9 mediated CD133^knockout^ cells in colony #1 and #2 showed decreased survivin expression, compared to the mock, GFP and CD133^+^ cells in colony #3. (GFP, CRISPR-Cas9 mediated control group; CD133 KO, CRISPR-Cas9 mediated CD133^knockout^ group).

We observed that the number of colonies in the CD133^knockout^ group was approximately one third that of the colonies in the mock and GFP groups (Fig 2A) (Mock *vs*. CD133^knockout^, P = 0.017; GFP *vs*. CD133^knockout^, P = 0.0172). A WST-1 assay revealed that the proliferation of CD133^knockout^ colon cancer cells were significantly inhibited at 24 h (P<0.001) compared with GFP group and mock group (Fig 2B). In western blot analysis, CD133^knockout^ cells showed decreased survivin compared with control (Fig 2C). The cleaved caspase-3 expression were increased in CD133^knockout^ cells in some colonies but not in all colonies. Therefore, the low number of colony in the CD133^knockout^ group may be associated with a decrease in cell proliferation rather than an increase in apoptosis.

### The effect of STAT3 inhibitor was dependent on CD133 expression in colon cancer cells

**Fig 3:**
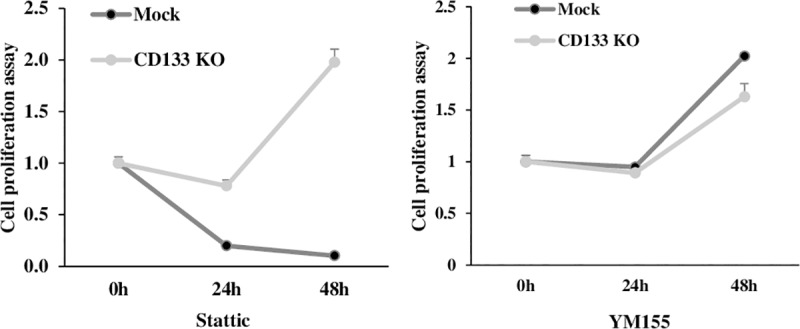
The stattic treatment induced much more inhibitory effect on cell proliferation of CD133^+^ cells than CD133^knockout^ cells (P<0.001). However, the effect of YM155 was not significantly different between CD133^+^ cells and CD133^knockout^ cells.

In WST-1 assay. the stattic treatment showed inhibition of cell proliferation in CD133^+^ cells in contrast to no effect on CD133^knockout^ cells (P<0.001) (Fig 3). However, the effect of YM155 showed no significant difference between CD133^+^ cells and CD133^knockout^ cells (Fig 3).

### Decreased cell migration and invasion with loss of vimentin expression in CD133^knockout^ colon cancer cells

**Fig 4:**
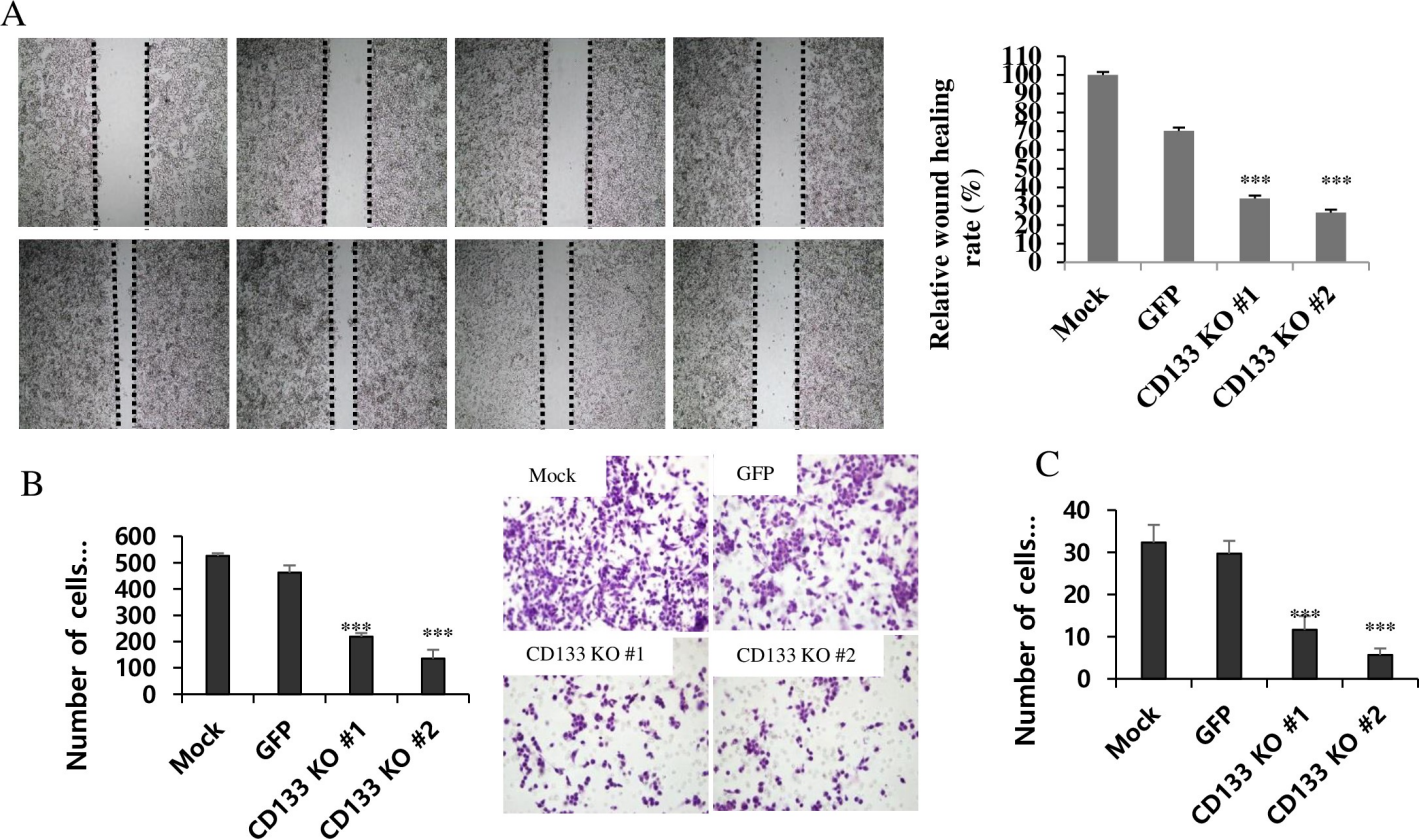
CD133 knockout inhibits cell migration and invasion. (**A**) Photographs at 0 and 24h after wounding demonstrated that CRISPR-Cas9 mediated CD133^knockout^ cells showed significantly decreased wound healing rate compared to the control (***P<0.001). (**B**) CD133^knockout^ cells had the least average number of migrating cells among the three groups in transwell migration assay (***P<0.001). (**C**) In transwell invasion assay, the average number of invasive cells in CD133^knockout^ group was 11.67 and control cells were 32.33 and 29.67, respectively (***P<0.001).

**Fig 5:**
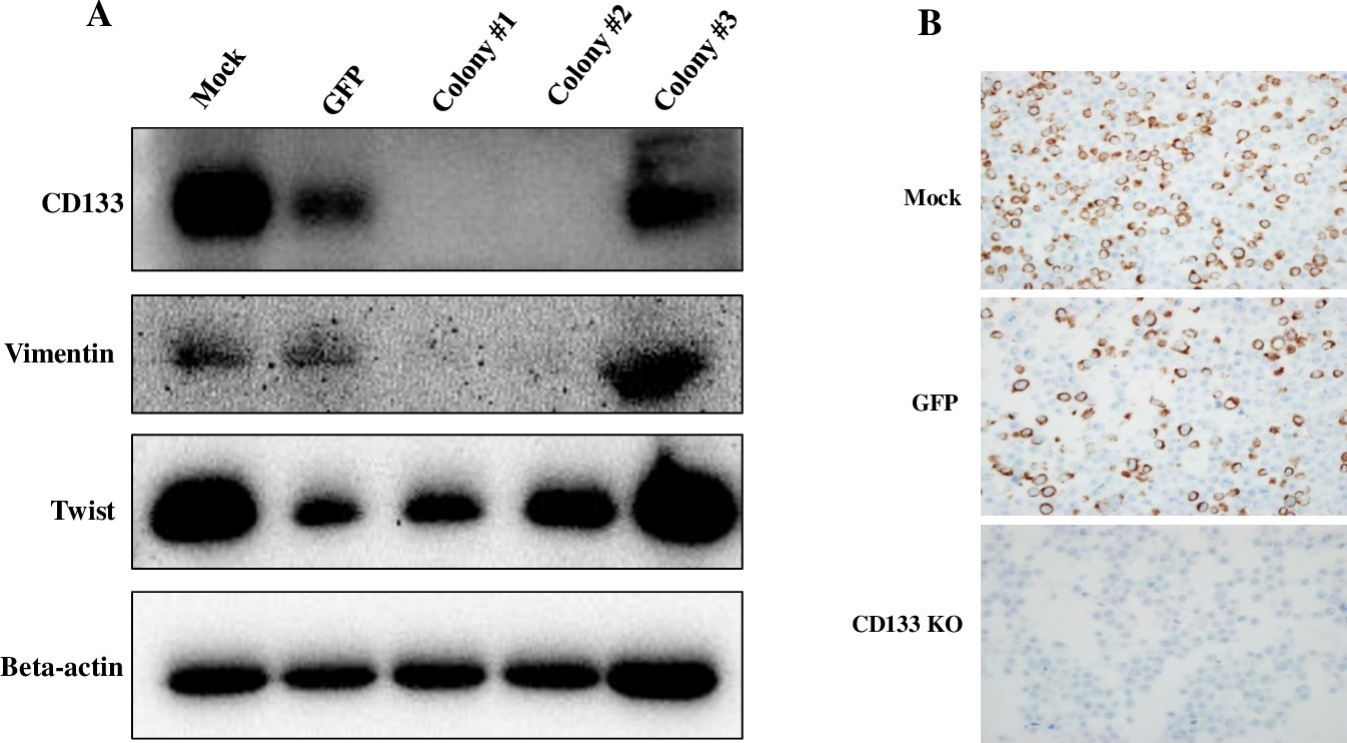
CD133^knockout^ cells showed loss of vimentin expression. (**A**) Among the EMT-related proteins examined in the current study, it is remarkable that vimentin expression has disappeared in CD133^knockout^ colon cancer cells in #1 and #2 colony incontrast to positive in CD133^+^ cells in #3 colony and Mock. (**B**) The immunohistochemical staining on cell blocks also showed loss of vimentin expression in CD133^knockout^ cells compared with control (mock and GFP).

In wound healing assay, the migration ability of CD133^knockout^ cells was significantly attenuated at 24 h after scratching (P<0.001, Fig 4A). In transwell migration assay, the average number of migrating cells in CD133^knockout^ group was 218.67 and control cells were 525.67 and 462.67 in the mock and GFP groups, respectively (P<0.001, Fig 4B). Transwell invasion assay also revealed significant inhibition of the invasive activities of CD133^knockout^ colon cancer cells (P<0.001, Fig 4C). The average number of invasive cells in CD133^knockout^ group was 11.67 and control cells were 32.33 and 29.67 in the mock and GFP groups, respectively. The western blot for the EMT-related proteins revealed loss of vimentin expression in CD133^knockout^ cells compared to control and CD133^+^ colony (Fig 5A). We also evaluated the E-cadherin, twist and CXCR4 expression, but they showed no significant difference. Loss of vimentin expression in CD133^knockout^ cells at protein level was also confirmed by IHC staining on cell blocks of CD133^knockout^, GFP and mock. (Fig 5B).

## Discussion

The CRISPR-Cas9 gene editing system is very useful in cancer cell biological research because it provides a method to modify gene expression at the DNA level in human cancer cell lines. CRISPR-Cas9 system guided gene targeting is highly specific and stable. In our previous report, we found CD133 was required for cell proliferation and biological behaviors of colon cancer cell via down-regulating CD133 expression by siRNA transfection [8, 9], but there was a limitation due to the transient inhibitory effect of siRNA. Moreover, knockdown using siRNA could not completely turn off the target genes, and the interventions of cells with residual gene function cannot be excluded. The potential side effects caused by ectopic RNA or transfection reagents could be effect on the experimental result. In this study, we generated a CD133^knockout^ colon cancer cell line by using the CRISPR-Cas9 gene editing system.

As expected, experimental results of CD133^knockout^ cells by CRISPR-Cas9 were similar to the results of our previous experiments with CD133-siRNA. We could get more consistent and convincing results because we were able to purify the CD133 knockout cells using the culture of a single cell colony. In spite of CD133 knockout, CD133^knockout^ cells were still made colony, but the colony number was remarkably decreased. Recently, we found activated signal transducer and activator of transcription 3 (STAT3) is upregulated by interleukin (IL)-6 and blocking of the STAT3/CD133/survivin signaling pathway showed anticancer effect in colon cancer *in vitro* [8, 10]. As observed in vitro result of the current study, the anticancer effect of stattic (STAT3 inhibitor) showed only in CD133^+^ cells (Fig 2C). These results confirmed our previous report, where activated STAT3 was shown to upregulate CD133 expression in colon cancer cells. However, the effect of YM155 was not dependent on CD133 expression.

We found CD133 knockout by CRISPR-Cas9 extensively inhibited cell migration and invasion of colon cancer cells (Fig 3A and 3B). Additionally, loss of vimentin expression was observed in CD133^knockout^ cells (Fig 4). Upregulation of mesenchymal markers, like vimentin, and loss of function of adherent junction protein E-cadherin are typical changes, indicating EMT of cancer cells [11]. CD133^+^ cells seemed to have more phenotypes toward mesenchymal cells in colon cancer progression [18]. We had reported that CD133 expression was common in the invasive part of adenocarcinoma, which is in contrast to its rare expression in adenoma in the surgically resected clinical sample [10, 19]. The previous studies reported that EMT contributed to the cancer cell invasion and metastasis in several malignant tumors, and several transcription factors which induce EMT might drive solid tumor progression, including colon cancer [20–22]. Zhang et al described that the induction of CXCR4^+^ expression by SDF-1 increased EMT and invasive behavior in cancer stem cells[18]. Recently, Dinicola *et al*. proved that triggering EMT by nicotine led to increased migration and invasion of colon cancer cells [23]. Vimentin has been known as an essential regulating factor of EMT and hypoxia-inducible facot-1 (HIF-1) has been described as a major transcriptional regulator of vimentin [24]. Decreased cell migration and invasion abilities might be related to loss of vimentin expression in CD133^knockout^ cells, as seen in the current study. These findings reflected that CD133 could play a critical role in tumor progression by regulating the expression of EMT-related molecules in colon cancer. Therefore, we propose further studies to investigate how CD133 and other factors interact with vimentin expression.

In conclusion, CD133^knockout^ cells showed remarkable inhibitory effects on tumorigenic property in colon cancer, including cell proliferation colony formation capacity, migration, and invasion. Therefore, modulating CD133 expression can be an effective treatment strategy for CD133^+^ colon cancer by inhibiting characteristics of cancer stem cells.
